# Opinion paper: artificial intelligence in cardio-oncology: a clinical call to action

**DOI:** 10.3389/fonc.2025.1662926

**Published:** 2025-08-12

**Authors:** Juliana Salas Segura, Esteban Zavaleta, Jeaustin Mora Jiménez, Kevin Cruz Mora

**Affiliations:** ^1^ Cardiology Department, Clínica Bíblica, San José, Costa Rica; ^2^ Health Research Department, Clínica Bíblica, San José, Costa Rica; ^3^ Pharmacy Department, Clínica Bíblica, San José, Costa Rica

**Keywords:** cardio-oncology, artificial intelligence, risk stratification, cardiovascular toxicity, machine learning models, remote monitoring

## Introduction

Cardio-oncology faces significant challenges, such as the high incidence of cardiovascular events in cancer patients, the heterogeneity of cardiotoxic manifestations, and the complexity of interdisciplinary management (1).

Current risk stratification tools, although widely used, present key limitations: low sensitivity in early stages, poor adaptability to different subgroups, and lack of international standardization, which hinders their consistent use in clinical practice and comparison across studies (1).

In the face of these diagnostic challenges in cardio-oncology, artificial intelligence emerges as a promising tool due to its ability to integrate clinical, electrocardiographic, imaging, and biomarker data. Preliminary studies suggest its potential utility for early detection of ventricular dysfunction and other cardiovascular adverse effects, even before they become evident through conventional methods (2).

## Current clinical applications of artificial intelligence in cardio-oncology

A real-world example of its applicability is its integration into cardiac magnetic resonance imaging and echocardiography, contributing to the detection of functional and structural abnormalities, which could facilitate earlier intervention and, in some cases, improve long-term clinical outcomes for patients (2).

Among the most promising advances is the use of AI models applied to baseline electrocardiograms to predict chemotherapy-related cardiac dysfunction (CTRCD). In patients treated with anthracyclines, an ECG-derived score has preliminarily shown greater predictive capacity than some traditional clinical models, allowing for the identification of at-risk patients before the onset of symptoms or echocardiographic deterioration (3). However, these results should be interpreted with caution, as they still require validation in larger cohorts and diverse clinical settings.

In the field of cardiac imaging, AI has demonstrated high accuracy in automating key parameters such as left ventricular ejection fraction (LVEF) and global longitudinal strain (GLS), both in echocardiography and cardiac magnetic resonance imaging (4).

These algorithms enable automatic segmentation, volume measurement, and reduction of interobserver variability, with results that are comparable to or even superior in reproducibility to traditional methods. This level of precision is particularly valuable in clinical settings where image quality is suboptimal or where close monitoring of ventricular function is required (4).

Risk scores developed using machine learning have shown high potential in predicting cardiotoxicity in oncology patients, by integrating biomarkers such as troponins and NT-proBNP along with clinical data, echocardiographic findings, and treatment characteristics (5). Although preliminary results are promising, the clinical implementation of AI still requires validation in real-world settings.

**Figure 1:**
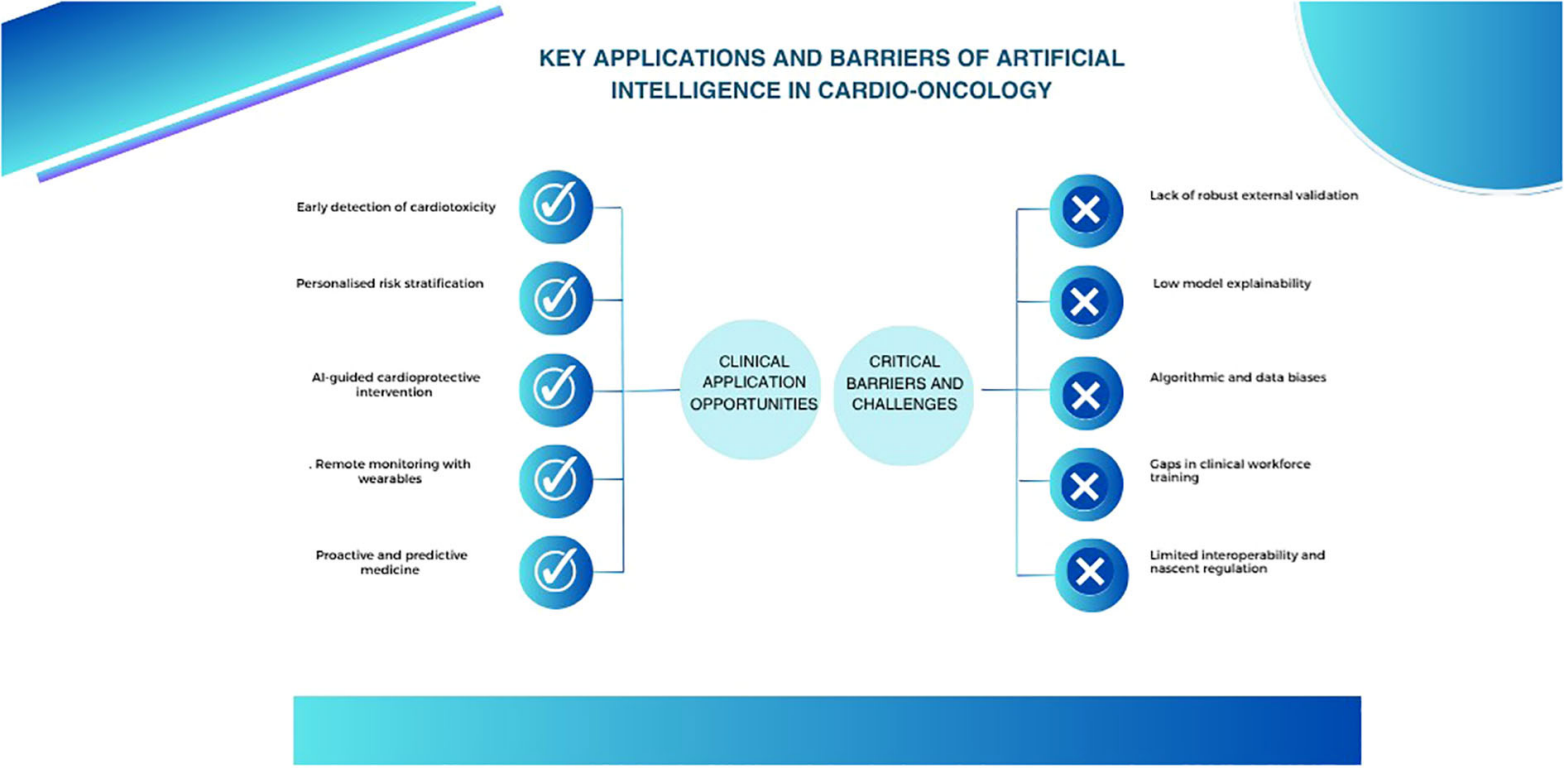
Artificial intelligence in cardio-oncology: clinical opportunities and critical barriers.

As shown in **Figure 1**, its widespread application is still limited by the lack of external validation, clinical training, and privacy safeguards, as well as by the heterogeneity in event definitions, variability in predictor selection, and data quality (2, 5).

Beyond its theoretical potential, as illustrated in **Figure 1**, artificial intelligence offers multiple tangible opportunities for integration into clinical cardio-oncology practice, particularly in settings where cardiovascular risk is high and rapid response capacity is essential.

A practical example of its applicability is evident in patients with breast cancer or lymphoma exposed to anthracyclines, where AI has shown potential to identify early those who will develop cardiotoxicity. Its use in surveillance strategies allows for action before clinical deterioration, optimizing follow-up and reducing the risk of therapy interruption (3).

Unlike traditional risk stratification models in cardio-oncology—which rely primarily on factors such as age, hypertension, diabetes, LVEF, and type of chemotherapy—AI models integrate multiple sources of clinical, imaging, and biomarker data, demonstrating superior performance in preliminary studies. In breast cancer, some models have reported AUCs >90%, outperforming the predictive capacity of conventional tools. However, clinical adoption still requires external validation and robust prospective studies (6).

Artificial intelligence has improved the accuracy of cardiovascular risk stratification in oncology patients, allowing the identification of subgroups with greater susceptibility to adverse events during and after treatment. This predictive capacity could facilitate the personalization of prevention strategies, such as adjusting oncologic treatment, intensifying monitoring, or the early initiation of cardioprotective therapies (7).

However, the direct use of AI to guide therapeutic interventions is still in the validation phase. Although clinical guidelines acknowledge its potential, they emphasize the need for prospective studies to demonstrate impact on decision-making and outcomes. This highlights the urgency of integrating these tools into collaborative multicenter studies (7).

## Wearables and remote cardiovascular monitoring in oncology patients

The use of wearable devices in oncology patients has attracted interest as a potential tool for remote monitoring, and their integration with artificial intelligence enables continuous physiological data to be transformed into clinically valuable signals. Variables such as heart rate, rhythm, estimated blood pressure, and sleep patterns can be recorded non-invasively and continuously, offering a dynamic overview of the patient’s cardiovascular status (8).

AI analyzes these signals in real time to identify subtle alterations associated with early cardiovascular events, even before symptoms become apparent. In the cardio-oncology context, this capability is especially relevant, as patients undergoing cardiotoxic therapies may develop arrhythmias, heart failure, or ischemia without evident clinical signs (8).

Machine learning algorithms applied to photoplethysmography and electrocardiogram data obtained from wearables have reported promising diagnostic performance in preliminary studies, achieving sensitivities and specificities greater than 90% for detecting arrhythmias such as atrial fibrillation. This enables not only early detection but also early intervention with potential prognostic impact, although clinical confirmation is still required (8).

In addition, some advanced systems can estimate blood pressure variations and detect early signs of hemodynamic deterioration by analyzing complex digital patterns. This technological development could allow for safer and more personalized longitudinal monitoring, which is especially valuable in the post-treatment stage of oncology patients at high cardiovascular risk (8).

## Ethical, technical, and structural barriers to AI implementation

Despite the enthusiasm surrounding its potential, the adoption of artificial intelligence in cardio-oncology faces critical ethical barriers. Data privacy, digital security, and informed consent remain vulnerable issues—particularly in settings where sensitive clinical records of oncology patients are handled. Patient protection must be a top priority from the design phase through to the implementation of any tool (9).

Another major challenge is algorithmic bias, often stemming from the use of datasets that do not reflect the true diversity of patients. This can lead to less accurate predictions for certain groups, perpetuating disparities in access to quality care. Mitigating this risk requires inclusive data selection and continuous review of algorithm performance (9).

Moreover, the lack of external validation in many models limits their generalizability. For AI to be truly reliable in clinical practice, it is essential to validate its results in independent populations and ensure that its predictions can be interpreted transparently. Only then can it earn the trust of both medical teams and patients (9).

From the perspective of oncology patients, artificial intelligence is viewed with moderate optimism, particularly regarding its potential to improve the quality of care. However, concerns persist about diagnostic errors, data privacy, and healthcare costs. These concerns are more common among individuals with lower educational levels, highlighting the importance of education and transparency for its effective adoption (10).

A recurring technical obstacle in the implementation of artificial intelligence models is the difficulty of translating their complex outputs into formats that are understandable and useful for clinicians. When healthcare professionals have limited time and are faced with low-transparency tools, it is natural for doubts to arise regarding their reliability. Therefore, it is essential that these technologies provide interpretable outputs and clear alerts about the limits of their applicability (9).

Moreover, advancing toward precision medicine in cardio-oncology cannot rely solely on developers or clinicians working in isolation. Ongoing collaboration among healthcare professionals, researchers, the technology industry, regulatory bodies, and reimbursement systems is essential. Only through this collective effort can AI-based solutions truly address the needs of both patients and healthcare teams (9).

The effective adoption of artificial intelligence in cardio-oncology demands cross-disciplinary collaboration among clinicians, engineers, data scientists, and medical informatics experts. Only through this joint effort can tools be developed that address real needs within the healthcare setting, maintaining clinical relevance from design through to implementation (8).

To foster acceptance among healthcare professionals, it is essential for AI models to be understandable. Algorithms must provide clear explanations of how their recommendations are generated, enabling clinicians to confidently interpret the results and identify potential errors or inconsistencies (8).

Another critical component is rigorous and continuous validation. Models must be evaluated in prospective clinical studies with external populations, comparing their performance against current standards. In addition, ongoing monitoring and regular updates are required to maintain accuracy amid clinical, technological, or population changes, as well as to ensure their applicability in future scenarios and diverse healthcare settings (8).

Equity must also be a foundational pillar in the development of these models. Considering social determinants and training algorithms on diverse populations is key to avoiding biases that perpetuate health disparities. This inclusivity enhances their applicability across different subgroups of oncology patients (8).

Finally, for the safe integration of AI into clinical practice, clear regulatory frameworks must be followed, such as those established by agencies like the FDA. This includes ensuring data privacy, establishing revalidation pathways when models are updated, and generating solid evidence to support their impact on clinical outcomes and the efficiency of the healthcare system (8).

The clinical implementation of artificial intelligence in cardio-oncology requires a structured roadmap that includes prospective validation in representative clinical cohorts, integration into electronic health systems, active participation of multidisciplinary teams, and training of clinical staff. It is also essential to establish mechanisms for monitoring, retraining, and oversight of the models, as well as strategies that ensure equity, transparency, and compliance with current regulatory frameworks (9).

## Discussion

Delaying the critical and responsible incorporation of AI into cardio-oncology means missing a valuable opportunity for clinical improvement. Artificial intelligence represents a paradigm shift in cardio-oncology by enabling earlier detection of cardiotoxicity, improved risk stratification, and continuous monitoring in vulnerable patients. These capabilities pave the way for a more personalized, proactive, and data-driven approach to medicine.

However, its clinical adoption still faces major barriers, such as the lack of external validation, limited model explainability, and the risk of bias in underrepresented populations. Moving forward requires the implementation of pilot projects, training of healthcare professionals, and the integration of equity principles from the design stage. More than a technological option, AI is an urgent clinical necessity.
